# Mechanisms Involved in the Association between Periodontitis and Complications in Pregnancy

**DOI:** 10.3389/fpubh.2014.00290

**Published:** 2015-01-29

**Authors:** Marcela Yang Hui Zi, Priscila Larcher Longo, Bruno Bueno-Silva, Marcia Pinto Alves Mayer

**Affiliations:** ^1^Department of Microbiology, Institute of Biomedical Sciences, University of São Paulo, São Paulo, Brazil

**Keywords:** periodontal diseases, pregnancy, preterm birth, inflammation

## Abstract

The association between periodontitis and some of the problems with pregnancy such as premature delivery, low weight at birth, and preeclampsia (PE) has been suggested. Nevertheless, epidemiological data have shown contradictory data, mainly due to differences in clinical parameters of periodontitis assessment. Furthermore, differences in microbial composition and immune response between aggressive and chronic periodontitis are not addressed by these epidemiological studies. We aimed to review the current data on the association between some of these problems with pregnancy and periodontitis, and the mechanisms underlying this association. Shifts in the microbial composition of the subgingival biofilm may occur during pregnancy, leading to a potentially more hazardous microbial community. Pregnancy is characterized by physiological immune tolerance. However, the infection leads to a shift in maternal immune response to a pathogenic pro-inflammatory response, with production of inflammatory cytokines and toxic products. In women with periodontitis, the infected periodontal tissues may act as reservoirs of bacteria and their products that can disseminate to the fetus-placenta unit. In severe periodontitis patients, the infection agents and their products are able to activate inflammatory signaling pathways locally and in extra-oral sites, including the placenta-fetal unit, which may not only induce preterm labor but also lead to PE and restrict intrauterine growth. Despite these evidences, the effectiveness of periodontal treatment in preventing gestational complications was still not established since it may be influenced by several factors such as severity of disease, composition of microbial community, treatment strategy, and period of treatment throughout pregnancy. This lack of scientific evidence does not exclude the need to control infection and inflammation in periodontitis patients during pregnancy, and treatment protocols should be validated.

## Introduction

Every year, about 15 million babies around the world are born preterm (PT) (after 23 weeks and before 37 weeks of gestation). Over 60% of PT births occur in Africa and South Asia, but PT birth is truly a global problem. Across 184 countries, the rate of PT birth ranges from 5 to 18% of the babies born and the highest rates per live births are presented in Malawi, Comoros, Congo, Zimbabwe, Equatorial Guinea, Gabon, Pakistan, Indonesia, and Mauritania (1). In Latin America, PT births comprise 7% of live births in Chile, 9% in Brazil, and reach 14% in Costa Rica, whereas in North America, it ranges from 8% in Canada to 12% in the USA. It is 7% in China. In Europe, it can be as low as 6% in Scandinavian countries, 7–9% in France, UK, Italy, and Germany, reaching 15% in Cyprus (2). Spontaneous PT labor leads to 70% of PT births, whereas the remainder is medically indicated because of maternal or fetal complications, such as preeclampsia (PE) or intrauterine growth restriction (3).

Babies born PT and/or those with intrauterine restricted growth conditions (IUGR) are usually born with low weight (LBW), which is considered as those born with <2,500 g (4). Although most studies use this reference value, it is deficient for PT birth. More recently, other generic references based on birth weight percentiles adapted to local populations were recently validated (5), but studies on the influence of different parameters using these new references are still lacking. A survey in 138 low-income and middle-income countries revealed that 22% of the children born in 2010 were both term and small-for-gestational-age, 8.1% were PT and appropriate-for-gestational-age, and 2.1% were PT and small-for-gestational-age (6).

Preterm birth is the leading cause of newborn deaths (in the first 4 weeks of life) (1). Low birth weight babies also present a higher risk of adverse outcomes in the perinatal period, including increased mortality rate (7). The mortality risk in the first month of life is 10–40 times higher for PT-small for the gestational age than for term and appropriate-for-gestational-age infants (8). Furthermore, babies born PT are at higher risk of developing neurological, respiratory, and gastrointestinal diseases, attributed to immaturity of multiple organ systems (3, 9). Many survivors face a lifetime of disability, including learning disabilities and visual and hearing problems (1).

Risk factors for small-for-gestational-age babies might differ from those to reduce the number of babies born PT. In South Asia, the prevalence of babies born at term but small for the gestation age is higher than in Latin America. The very high rates of small for the gestational age babies might be explained by higher rates of adolescent pregnancy, chronic maternal malnutrition, low pre-pregnancy body-mass index, low weight gain in pregnancy, and low maternal height (8). The prevalence of LBW and PT is usually higher in low-income populations, however, other factors besides the socioeconomically status and access to prenatal health care (10), also influence these rates. Other common causes of PT birth include genetic and ethnic factors, multiple pregnancies, age, behavioral, and environmental factors including tobacco usage, chronic conditions (such as diabetes and high blood pressure), and infections (1, 11).

One of the most common and dangerous complications of pregnancy is PE. It is a multisystem disorder associated with elevated blood pressure and proteinuria, typically after 20 weeks of gestation. PE is primarily a generalized dysfunction of the maternal endothelium, which appears to be part of an exaggerated systemic inflammatory *response* that involves maternal leukocytes and pro-inflammatory cytokines. Briefly, PE is the result of inadequate development of maternal spiral arteries due to insufficient trophoblast invasion, leading to narrower blood vessels, which limit the blood supply from the mother into placental blood spaces. Acute arthrosis of spiral arteries aggravates the problem, resulting in hypoxia of the placenta. In a second stage, the ischemic placenta sheds subcellular trophoblast microparticles *to the maternal circulation*, which induces maternal leukocytes and endothelial cells to produce pro-inflammatory cytokines. Worldwide, PE affects an estimated 2–10% of pregnant women and is the chief cause of induced PT delivery, and slow growth of infants (12).

Even at very early gestation, fetal cells leak and are found in maternal circulation. The embryo/fetus expresses paternal antigens, which have to be tolerated throughout the gestation period by the mother (13). Thus, the maternal immune regulatory system must be activated in order to suppress maternal immunity toward fetal antigens, to allow implantation and fetus development during gestation, but still being able to elicit normal immune responses against infections (13, 14).

Regular pregnancy is predominantly characterized by a Th2 type immune response, which determines the protective maternal response to fetal development (15–18). Hormones, such as progesterone and prostaglandin E, play a key role in this Th1–Th2 shift during pregnancy (14, 19). More recently, the Th1/Th2 paradigm has been expanded with the addition of Th17 and regulatory T-cell (Tregs). The Th-17 inflammatory response is also dampened during pregnancy (20) and Tregs play a key role in maternal tolerance. Tregs recruitment and expansion are induced even before conception by the seminal fluid (21) and then by the embryonic trophoblasts, and these cells display immunosuppressive features, promote invasiveness, result in preferential production of Th2 cytokines and reduce the cytotoxicity of decidual natural killer (NK) cells (22).

Inflammation breaks the homeostasis at the maternal–fetal interface, revoking these immune privileges during gestation (9) and induces a Th1 pattern of cytokines, with significant fetal losses (23). Certain infectious diseases, even sub-clinical infections, may result in enhanced IL-12 production and in turn an overall shift toward type 1 bias. Th-1 response leads to the activation of decidual macrophages, which secrete toxic levels of nitric oxide and TNF-α. This leads to maternal rejection of the implanted embryo, which is termed as cytokine-triggered vascular autoamputation and involves the activation of coagulation mechanisms, resulting in vasculitis affecting the maternal blood supply to the implanted embryo (19). Furthermore, extracellular bacterial or fungal pathogens elicit a Th17 response, and an imbalance in the proportion of Th17/Treg is associated with recurrent pregnancy loss and other gestation adverse effects (13).

Infections during pregnancy, caused by bacteria, viruses, and parasites, can lead not only to PT birth but also to fetal death, injury, or other organ sequel depending on the pathogen. Some pathogens are known as classic teratogens, designated as TORCH (*Toxoplasma gondii*, *Treponema pallidum*, rubella, cytomegalovirus, and herpes simplex virus), but others may be included in this list, such as parvovirus B19, varicella zoster virus, and *Plasmodium falciparum* (24).

About 50% of the PT births were not associated with any known factor (11), leading to the search of alternative factors. Maternal infections of the genitourinary tract may promote the migration of cervical vaginal bacteria to the uterus (25). Bacteria colonizing the vagina, such as *Escherichia coli*, *Gardnerella vaginalis*, group B *Streptococcus*, and *Mycoplasma hominis* may ascend from the lower genital tract and were recovered in the amniotic fluid (24) and placenta (9). Infections in other parts of the body may also play an important role in the induction of births and prematurity by stimulating an immune response and due to the transit of microorganisms and/or their toxins in the bloodstream to the maternal–fetal unit (26).

Thus, differing from the common thinking not too long ago, the fetal-placenta unit may not be sterile, and nearly one-third of placental specimens harbor intracellular bacteria in the basal plate (the tissue layer at and below the maternal–fetal interface). Furthermore, the prevalence of placenta samples with detectable bacteria is higher in PT deliveries, although most of these infections are asymptomatic (27). Also contradicting previous paradigms, a recent placenta microbiome study reported a very close similarity between placental and oral microbiomes, and not with the urogenital tract microbiome, as expected (28). This latter study has also revealed a correlation between the placental microbiome with PT birth.

In this context, the infected periodontal tissues may be associated with prematurity by acting as reservoirs of bacteria and their products, which can disseminate to the fetus-placenta unit. Furthermore, immunological mediators at high concentrations produced locally at the infected gingival tissues or systemically can reach the fetus-placenta unit, resulting in prematurity and low weight at birth (11, 29). Although some aspects of the association between periodontitis and complications in pregnancy were elucidated, this relationship needs to be better studied and characterized. Therefore, we aimed to review the existing literature associating periodontal disease, its microbiota and immune response with the complications in pregnancy.

## Pregnancy and Periodontal Diseases

Periodontitis is an inflammatory disease of the soft and hard support tissues of tooth in response to the supra and subgingival microorganisms. It is classically divided into chronic and aggressive periodontitis (30). Chronic periodontitis is characterized by a slow and continuous destruction of periodontal tissues (31, 32), while the destruction of the periodontal ligament and alveolar bone is more rapid and severe in aggressive periodontitis (33). Furthermore, periodontitis can affect most teeth (generalized), or can be restricted to a group of teeth, and described as localized.

Despite the enormous microbial diversity in the oral cavity, up to now, few species were associated with periodontitis (34), based mainly on their higher prevalence in diseased subjects, and their elimination after a successful treatment. The Gram negative anaerobes of the Socransky’s red complex, *Porphyromonas gingivalis*, *Treponema denticola*, and *Tannerella forsythia* (35) have been associated with chronic periodontitis (36). *P. gingivalis*, *T. denticola*, *T. forsythia*, and the facultative Gram negative rod *Aggregatibacter actinomycetemcomitans*, particularly serotype b, and to a lesser extent serotype c, were found in higher levels in patients with aggressive generalized or localized periodontitis when compared to controls (37, 38). However, patients with localized disease did not present high IgG titers to *T. forsythia* as observed for patients with generalized periodontitis, although both were colonized by the organism, suggesting differences in immune response associated with the extension of disease (39).

Recent data suggested an additional list of suspected pathogens formed by 17 species or phylotypes, mostly detected by high throughput sequencing techniques, but more studies on their *in vivo* ecological roles are needed before they can be recognized as periodontopathogens (40). Despite the dramatic differences in the microbial composition in healthy periodontium and periodontitis, periodontal pathogens may not directly cause periodontitis but rather they redesign the typically symbiotic microbiota into a dysbiotic one, which disrupts the normal homeostatic relationship with the host tissues (41). Thus, not only would certain pathogens be involved in the disease but rather a dysbiotic community may challenge the host, and the inflammatory destructive process is the result of these multiple interactions.

The response to a microbial challenge is not homogeneous in man, and several polymorphisms have been reported in genes associated with the production of cytokines. Some of these polymorphisms have been associated with increased susceptibility to periodontitis in certain populations (42).

The subgingival microbiota and host susceptibility differ between aggressive and chronic periodontitis, leading to differences in cytokines and adhesion molecules (43). The local cytokines production in gingival tissues differs between types of periodontitis, although no differences in inflammatory mediators in serum were demonstrated (44). Gingival tissues from patients with aggressive periodontitis present higher levels of protein-1-α (MIP-1α), IFN-γ-induced protein 10 (IP-10) and its receptors CCR5 and CXCR3, and lower levels of IL-10 than tissues of chronic periodontitis patients. On the other hand, tissues of chronic periodontitis show increased expression of monocyte chemotactic protein 1 (MCP-1) and its receptor CCR4 (45).

Likewise, the levels of the adhesion molecule CD11a of T cells also differ in tissues from aggressive and chronic periodontitis, evidencing distinct cellular sources of immune regulatory cytokines (46). The levels of the pro-inflammatory cytokine IL-17 in gingival crevicular fluid (GCF) of aggressive periodontitis are higher than in GCF of chronic periodontitis patients, whereas the levels of IL-11, an anti-inflammatory mediator, were decreased in aggressive compared to chronic periodontitis (47). Interestingly, IL-17 induces the production of prostaglandin E2 (PGE2), and is associated with increased bone loss (48), whereas IL-11 induced osteoblastic differentiation and bone formation (49).

Not only may periodontitis interfere with pregnancy but also pregnancy may alter the progression of periodontal diseases (PD). Physiological changes induced during pregnancy may alter the inflammatory response, amplifying the gingival inflammation. Pregnancy gingivitis affects 36–100% of pregnant women (50). Clinical parameters such bleeding on probing and pocket depth (PD) may increase during pregnancy, without concomitant increase in plaque index, which decreases after delivery (51). The mechanisms underlying the increased severity of PD during pregnancy were associated to increased vascular permeability, depression of the immune system, and shifts on the composition of supra and subgingival microbiota (50).

Early studies have demonstrated that *Prevotella intermedia* and, to a lesser extent, *P. gingivalis* can use progesterone and estradiol in replacement of vitamin K, a required growth factor for these species (52). More recently, an increase in salivary progesterone concentration from the first to the second trimester of pregnancy has been linked to increased levels of *P. gingivalis* (50).

Culture and target molecular techniques for bacterial identification have been used to evaluate the effect of gestation on the oral microbiota. The increase in clinical parameters of periodontal disease severity, such as increased bleeding on probing during gestation, is associated with increased levels of anaerobic species associated with mature dental biofilm including species of *Fusobacterium* and *Prevotella*, *Streptococcus anginosus*, and *Streptococcus intermedius* (53). Other data reported that the proportions of certain putative periodontopathogens such as *A. actinomycetemcomitans* and *Parvimonas micra* were increased during pregnancy (50). In a case–control study, levels of eight oral bacteria species (including the red complex and *A. actinomycetemcomitans*) tended to increase from 22nd week of pregnancy in mothers of premature babies, while these levels remained stable in mothers at term delivery. The prevalence of *A. actinomycetemcomitans* was very low in the PT group during early pregnancy, but increased during pregnancy, reaching levels 2.42-folds higher in the PT group after delivery than in the term group (54).

The microbial diversity of the oral cavity has still not been evaluated in a longitudinal study throughout the gestational period. However, metagenomic studies on the gut microbiota of pregnant women may provide some clues on the microbial shifts occurring in mucosa surfaces over the course of gestation. Metagenomic analyses of stools of pregnant women in different gestational phases and controls demonstrated an increase in abundance of Proteobacteria and Actinobacteria from the first to the third semester, and a decrease in microbial richness, which persisted 1 month postpartum. Moreover, the microbial shift was followed by increased levels of IFN-γ, IL-2, IL-6, and TNF-α in stools, indicating that despite the anti-inflammatory conditions at the placental interface, gestation leads to low-grade inflammation of the intestinal mucosa. It is important to notice that the microbial composition determined the inflammatory status of the mucosal surface, and not the opposite, since the experimental transfer of the fecal microbiota obtained from women at the third semester of gestation (fecal transplantation) was able to induce inflammation in recipient mice (55). These data evidenced that changes in immunity and/or hormone levels during gestation induce changes in the composition of the microbiota, which leads to increased inflammatory response in mucosal surfaces.

Altogether, these data provide evidences that physiological changes associated with pregnancy lead to shifts in the microbial communities colonizing mucosa surfaces, which then induce pro-inflammatory immune responses. Thus, the already dysbiotic microbiota in subgingival sites of PD patients would be submitted to additional factors promoting imbalance during pregnancy, increasing its pathogenic potential to induce gingival inflammation.

Epidemiological data suggested that PD represent a potential risk for PT birth, as shown in Table 1. Cross-sectional studies have shown the association between PD and prematurity and/or low birth weight (56, 57). Case–control studies indicated that periodontitis can increase the risk for prematurity by seven times (58). On the other hand, two case–control studies carried out in Brazil (59, 60) showed no relationship between periodontitis and PT low birth weight.

**Table 1:** **Epidemiological data on the association of periodontal diseases and preterm birth and/or low birth weight and other alterations of the gestational pattern**.

Study type	Participants number	Parameters	Results	Reference
Case–control	Controls *n* = 124 Cases *n* = 93	LBW, MPD, PTB, preterm labor, premature rupture of membranes	LBW significantly high in mothers with more severe PD, indicating that MPD is a significant risk factor for LBW	Offenbacher et al. (61)
Case–control	Controls *n* = 611 Cases *n* = 304	IUGR, LBW, MPD, PTB	Results do not support the hypothesis of association of MPD and IUGR, LBW, and PTB	Bassani et al. (59)
Case–control	Control: *n* = 1042 IUGR *n* = 77 LBW *n* = 235 PTB *n* = 238	IUGR, LBW, MPD, PTB	MPD is associated with an increased risk for PTB, LBW, and IUGR	Siqueira et al. (57)
Case–control	Controls *n* = 393 LBW *n* = 96 PTB *n* = 110 PTB + LBW *n* = 63	LBW, MPD LBW, PTB + LBW	MPD was more severe in control individuals than in cases. The extent of periodontal disease did not increase risk of LBW. Mean PPD and frequency of periodontal sites with clinical attachment level ≥3 mm in PTB + LBW cases were lower than in control	Vettore et al. (60)
Case–control	Controls *n* = 20 PE *n* = 20	MPD, PE	PE was associated with MPD. PE cases were 4.33 times more likely to have MPD (OR = 4.33)	Varshney and Gautam (62)
Case–control	Controls *n* = 44 LBW *n* = 44	Bleeding on probing, presence of supragingival calculus and CPITN (community periodontal index for treatment needs), LBW, MPD	MPD was associated with LBW. Mothers of LBW infants had less healthy areas of gingiva and more deep pockets	Haerian-Ardakani et al. (63)
Cohort	*n* = 1,115	PE, PD	Women were at higher risk for preeclampsia if they had severe periodontal disease at delivery (OR = 2.4), or if they had periodontal disease progression during pregnancy (OR = 2.1)	Boggess et al. (64)
Cross-sectional	*n* = 449	LBW, MPD, PTB, LBW + PTB	There was no statistically significant association between MPD and LBW	Lunardelli and Peres (56)
Cross-sectional	*n* = 277	LBW, MPD, PE, PTB	MPD were at higher risk for developing PE, PTB, and LBW. The rate of PE in women with PD was 18.6% compared to 7.3% in the control group (OR = 2.7). The OR for PTB was (4.4) and for LBW was (3.5) when mother had PD	Alchalabi et al. (65)
Cross-sectional	*n* = 770	LWB, MPD, PPD, PTB, reduced maternal haemomglobin levels	Mothers with PPD >6 mm (OR = 2.21) had a higher risk of LBW. The increase in the severity of MPD was associated with increased in PTB and the MPD severity influenced the maternal hemoglobin levels, i.e., more severe periodontitis was associated with lower hemoglobin levels	Kothiwale et al. (66)
Cross-sectional	*n* = 390	Periodontopathogens, MPD	Prevalence of gingivitis was 38% and clinical periodontitis was 10%. Among the periodontitis group, high detection rates of *P. gingivalis* (56%), *Prevotella nigrescens* (44%), *T. denticola* (32%), and *P. intermedia* (24%) were associated with PD	Tellapragada et al. (67)
Prospective study	*n* = 1017	PD, LBW	Moderate or severe periodontal disease was associated with a LBW, a risk ratio of 2.3 (1.1–4.7), adjusted for age, smoking, drugs, marital and insurance status, and preeclampsia. Moderate or severe periodontal disease early in pregnancy is also associated with LBW	Boggess et al. (64)
Prospective study	*n* = 283	PD, PE	There was a significant relationship between periodontitis and the occurrence of preeclampsia among never-smokers (OR = 5.56)	Ha et al. (68)

*IUGR, intrauterine growth restriction; LBW, low birth weight (<2,500 g); MPD, maternal periodontal disease; PD, periodontal disease; PE, preeclampsia; PPD, probing pocket depth; PTB, preterm birth (gestacional age <37 weeks)*.

This review did not aim to cover all epidemiological studies on the topic but rather to discuss the reasons of conflicting results. This is covered by several systematic reviews with meta-analyses, which revealed a significant risk of PT delivery and low birth weight with periodontitis (69–71). The odds ratio for PT birth in mothers with periodontitis varied from 1.7 to 2.73; for low birth weight from 1.5 to 2.11; and for PT birth and low weight, from 2.35 to 3.57. Therefore, all meta-analysis demonstrated that PD could promote adverse pregnancy outcomes. In addition, meta-analyses (72, 73) have also identified a significant association between PE and PD.

As shown in Table 1, the results of some cohort, case–control, prospective, and cross-sectional studies were conflicting due to factors as number and demographic characteristics of the participants, parameters for periodontal disease diagnosis, inclusion of pregnant women in different gestation periods, and different statistical analysis.

Most studies on the association between periodontitis and gestational complications considered the subjects either as diseased or healthy. However, the incidence of PT delivery increases with increased severity of periodontitis (74, 75). Furthermore, few studies correlated the outcome in gestation with microbial parameters (Table 1). Due to variations in the microbial composition and immune response among periodontitis patients, it seems clear that the severity, extension, and type of periodontitis should be considered when the systemic effects of periodontitis are evaluated in a population. Environmental and genetic factors involved in altered immune response (42) against bacterial infections may also influence the effect of periodontitis in pregnancy.

These confounder factors prevented a definitive conclusion whether periodontitis is an independent risk factor of PT birth and/or low birth weight or PE, and larger randomized controlled trials to explore causality and to dissect the biological mechanisms involved in the association of periodontitis and gestation outcomes are still needed.

## Dissemination of Oral Bacteria and Their Products to Extra-Oral Sites

The effect of periodontal pathogens in distant sites is related not only with prematurity and low birth weight babies but also with other systemic diseases such as arthritis, cardiovascular disease, atherosclerosis, liver disease, diabetes, stroke (16), and Alzheimer’s disease (76). Oral bacteria are frequently found infecting extra-oral sites. *S. intermedius* was reported causing a brain abscess (77). *A. actinomycetemcomitans* is the most prevalent species in bacterial endocarditis among members of the HACEK group (*Haemophilus*, *Aggregatibacter*, *Cardiobacterium*, *Eikenella*, and *Kingella*), which includes the facultative anaerobic Gram negative bacilli that inhabit the oropharynx associated with endocarditis (78). *P. gingivalis* (79) and *A. actinomycetemcomitans* (80) specific DNA could be detected in atherosclerotic plaques in the aorta.

Several species present in the gastrointestinal tract and oral cavity were detected in neonatal gastric aspirates (81). The oral origin of most periodontal pathogens such as *A. actinomycetemcomitans*, *P. gingivalis*, and *T. forsythia* found in extra-oral sites is highly possible since they inhabit the oral and no other mucosal surfaces such as the vagina and intestine. On the other hand, *Fusobacterium nucleatum* is associated not only with oral but also with vaginal mucosa membranes (82, 83). However, the oral origin of *F. nucleatum* in the placenta-fetal unit has been considered since this species was detected in samples obtained from neonatal gastric aspirates, only of mothers with periodontal pockets (83) and the same strain was not present in the vaginal samples (84).

Analyses of amniotic fluid or placenta samples have demonstrated the presence of several oral bacteria, mainly *P. gingivalis* and *F. nucleatum* (85–88). *P. gingivalis* was detected in the amniotic fluid from mothers with premature labor and periodontitis. Subsequently, this periodontal pathogen was detected in placenta, in chorionic trophoblasts, and in several types of cells such as amniotic epithelial, decidual, and vascular (87).

*Fusobacterium nucleatum* is the most frequently detected species in the amniotic fluid of premature births with intact membranes (89, 90). In addition, *F. nucleatum* was linked to early-onset neonatal sepsis once it was detected in cord blood and amniotic samples from PT births (91–93).

Periodontal pathogens, especially *P. gingivalis* and *A. actinomycetemcomitans*, were detected in the amniotic fluid of pregnant women with periodontitis (94). These periodontal pathogens were also related to placenta infections in hypertensive women (95).

Patients with PE showed higher levels of *T. forsythia*, *F. nucleatum*, and *A. actinomycetemcomitans* in placenta. In addition, 50% of placenta samples from women with PE were positive for one or more periodontal pathogens while only 14.3% of the control group samples had detectable levels of periodontopathogens. Moreover, infection levels were significantly higher in the PE group (86).

Recently, the placental microbiome was characterized in a population-based cohort study with placenta samples from 320 subjects (28). Interestingly, a very close similarity between placental and oral microbiomes was demonstrated, and not with other niches such as the genitourinary tract or the gut. However, only one patient diagnosed with periodontitis was enrolled in that study, thus no association between the microbial composition of placenta and periodontitis could be made.

Present data support the hypothesis that oral bacteria can reach the maternal–fetal unit and may lead to gestational alterations. However, whether the colonization of commensal or putative periodontopathogens would result in different outcomes in pregnancy remains to be elucidated. Further studies, using new generation sequencing techniques, must be performed to evaluate the diversity of the microbiota at the placenta-fetal unit and mucosal surfaces, including the oral cavity, in order to establish the association of specific microbial communities with gestation alterations.

## Mechanisms Involved in the Association between Infections and Preterm Birth and Other Gestational Complications

The inflammatory cascade triggered by bacterial infections is supposed to play a central role in the pathogenesis associated with premature birth and fetal injury. Bacterial products stimulate the production of cytokines by placental tissues, as chorion, decidua, and trophoblasts (96). During infection, the cytokines produced by the fetal membranes in response to bacterial pathogens have a dual role: to control the growth of bacterial agents and, at the same time, to exacerbate the inflammatory process, which may lead to fetal injury and premature labor (9).

Generally, there is a relationship between the level of periodontal pathogens colonizing the subgingival sites and the antibody response to these pathogens (38, 39). However, the data on the relationship between maternal antibody response to periodontal pathogens and low weight at birth are contradictory.

Levels of maternal IgG against oral organisms were inversely related to premature and low weight births, suggesting that the antibodies would offer protection to the mother and fetus (97). In contrast, women in the second trimester of pregnancy with high serum IgG levels to *P. gingivalis* were more prone to have low weight babies than those with normal values (98). On the other hand, pregnant women with periodontitis and low titers of anti-*P. gingivalis* antibodies were seven times more at risk of PT delivery than those with high titers, suggesting that the suppression of maternal IgG response to *P. gingivalis* would be a risk factor associated with PT delivery (54). Furthermore, the levels of antibodies to *P. gingivalis* were 30% higher in placenta tissue of PT births due to chorioamnionitis, than in those at term delivery (87). Overall, the data suggest that high antibodies titers to *P. gingivalis* are usually indicative of periodontal pockets with high levels of this organism, leading to increased risk for PT birth. However, in heavily colonized women (severe periodontitis), when the antibodies response to periodontopathogens is low, the risk of gestation alterations would be higher than in women with high antibodies titers.

Pathogens and their products are recognized by receptors of the innate immune system, and the binding of these evolutionarily conserved recognition molecular patterns (PAMPS) to their receptors activates several signaling pathways, leading to an inflammatory response aiming to eliminate the bacterial agent. Each receptor located on the cell surface of various cells of the human body is specific for the recognition of a PAMP. For instance, the lipopolysaccharide (LPS), located at the outer membrane of Gram negative bacteria, is recognized by a toll-like receptor (TLR), mainly TLR4.

As described in the previous sections, the microbial community in subgingival sites of periodontitis patients is formed by a high percentage of Gram negative bacteria, of which the LPS is continuously challenging the host cells. Thus, several studies that reported the effect of LPS on pregnancy could help explaining the role of the microbial community on gestational complications. However, it is worth mentioning that LPS from different bacteria may exhibit different properties. For instance, *P. gingivalis* presents a complex atypical LPS that is not only recognized by TLR4 but also by TLR2, resulting in different signaling than that promoted by TLR4 binding (99). On the other hand, *P. gingivalis* represents a very low proportion of the subgingival microbiota, despite its association with periodontitis, and both TLR4 and TLR2 are highly expressed in inflamed gingival tissues (100). Thus, the effects of *E. coli* LPS, a TLR4 binding LPS, as described next, may not be interpreted as the effects of the whole LPS of subgingival sites.

Interestingly, the expression of TLR4 in placenta is higher in women with premature delivery and chorioamnionitis than in controls (101). LPS induces the production of pro-inflammatory mediators such as prostaglandins, which are modulators of uterine contractions. In addition, LPS stimulation of uterine tissues in mice with NK cell deficiency showed a reduction in the production of Th1 cytokines. This indicates that the NK cells of the uterus, which produce cytokines in the early stages of pregnancy, can be cytotoxic in the presence of pathogens, by producing pro-inflammatory cytokines such as TNF-α and IL1-β, and leading to deleterious effects to the fetus such as low placental and fetal weight (102). It is important to notice that the LPS dose tested in experimental mice in the latter study was 250 times lower than used for the endotoxemic shock, thus it would possibly simulate the LPS produced during a non-lethal infection by a Gram negative bacteria.

An *in vivo* model of PE demonstrated that infusions with LPS in the first and 14th day of gestation induced increased blood pressure and proteinuria (103). Furthermore, the LPS-induced PE led to disseminated intravascular coagulation (104).

Systemically, periodontitis results in higher levels of several inflammatory mediators, such as IL-6 and TNF-α, in response to the challenge promoted by the oral biofilm (105). Experimental intravenous injection of IL-6 in pregnant rats revealed that this cytokine can directly induce maternal fetal injury and stimulate the release of fetal hormones that cause stress (106).

TNF-α is a well-recognized mediator of activation of endothelial injury, a key mechanism of pathogenicity of PE. Blood cells of patients with severe PE produce higher levels of TNF-α without any stimulus. Nevertheless, LPS stimulus reduced the release of TNF-α by these cells indicating that the spontaneous release of TNF-α by leukocytes from patients with PE is the result of the disease process (107).

However, high concentrations of LPS can modulate myometrium contraction, and then induce PT delivery, possibly due to increased levels of IL-6, TNF-α, and PGF2. After treatment with LPS, additional TNF-α promotes even more contraction of myometrium cells (108).

Furthermore, an experimental animal model showed that fetal exposure to LPS can alter the development and permeability of intestinal epithelium (109), resulting in increased necrotic lesion (110). The fetal ingestion of amniotic fluid with microorganisms and microbial products such as LPS can cause enteritis, accelerate the motility of the colon, and results in the production of meconium during intrauterine life (111). Fetal exposure to intrauterine infection was also associated with fetal inflammatory response syndrome, leading to intrauterine growth restriction (112).

Studies in experimental animal models evaluated the effects of few bacteria species or their products in gestation, and definitively should not be extrapolated to the effects induced by the whole oral microbiota. However, they evidenced that the characteristics of the prenatal infecting pathogen may determine the outcome of infection in the development of pregnancy. For instance, the challenge of human fetal membranes with *G. vaginalis* and *Candida albicans*, but not with *Streptococcus agalactiae*, results in production of IL-10, which acts essentially in maintaining pregnancy by limiting the damaging effects of pro-inflammatory cytokines (113).

The intravenous inoculation of different *P. gingivalis* strains in rats on the 14th day of gestation, led to the placenta colonization at the 18th day. Interestingly, placenta colonization was dependent on the *P. gingivalis* strain, with a higher virulence potential of the fimbriated ones (114). Lately, infusion of *P. gingivalis* LPS on the 14th day of gestation in pregnant rats resulted in increased maternal systolic pressure, reduced placental weight, decreased fetal weight, and induced fetal resorption without causing generalized inflammatory response (115).

The intravenous inoculation of *F. nucleatum* into pregnant rats resulted in infection of placental membrane and invasion of the amniotic cavity, causing premature births, stillborn fetus, and shorter survival (116). Pregnant rats infected with *F. nucleatum* showed increased expression of IL-8 in a TLR-dependent manner. Furthermore, TLR4A (TLR4 agonist), but not TLR2A, reduced fetal death. Interestingly, TLR4 deficiency led to a reduction in the inflammatory response without affecting bacterial colonization in placenta, suggesting a possible use of TLR4 agonists as co-adjuvant of antibiotic therapy in the treatment of women with Gram negative bacterial infections during pregnancy (90).

On the other hand, intravenous inoculation of dental plaque containing *P. gingivalis* and *F. nucleatum* resulted in the presence of *F. nucleatum*, but not of *P. gingivalis* in placenta (88). This data should be taken with caution since the levels of both species possibly differed in the inoculums, and *P. gingivalis* exhibits strain with different virulent potential.

The subcutaneous inoculation of viable or heat-killed (HK) *P. gingivalis* in hamsters on the 8th day of gestation resulted in increased production of PGE2 and TNF-α. In animals inoculated with HK bacteria, the fetal weight was reduced in 24% and fetal resorption was 10.6% more frequent than in the control (117). Moreover, an experimental model in pregnant mice where viable *P. gingivalis* cells were inoculated into subcutaneous cameras resulted in the presence of the bacteria in the uterus and liver of infected animals, and fetus were small for the gestational age (118).

Our research group showed that rats infected subcutaneously with *P. gingivalis* at different stages of gestation showed elevated levels of IL-6 and TNF-α in serum and placenta, lower maternal weight gain, and low fetal weight when compared to non-infected controls. The infectious process was more intense and widespread in rats infected before or in the middle of the pregnancy when compared to groups infected at the beginning of pregnancy. Fetus-placental resorptions were observed mainly in mid-term pregnant infected rats, indicating that infection with *P. gingivalis* interferes more severely in fetus when the infection occurs at this phase of pregnancy (119).

Periodontitis induced by ligature in baboons provided evidence of a correlation between anti-*P. gingivalis* antibodies and pregnancy alterations (120). Periodontitis induced at the end of pregnancy resulted in increased production of IL-6, while the levels of PGE2 and IPB (induction factor of bactericidal permeability) increased only in groups that received ligature before pregnancy or in the 3rd month of pregnancy (121).

Despite the value of these results, these trials do not characterize chronic disease observed in humans. In general, these studies suggest that increased synthesis of inflammatory cytokines and metaloproteinases promoted by periodontopathogens could induce premature labor. Moreover, the translation of data obtained in animal models to humans may also be jeopardized by differences in placenta and uterus structure, hormones regulation, immune system, length of gestation between other mammals and humans (122). However, they are valuable evidences of the role of bacteria and their products in affecting the homeostasis of the maternal–fetal unit and leading to abnormal pregnancy.

The current understanding of PT labor is that the switch of the myometrium from a quiescent to a contractile state is accompanied by a shift in signaling from anti-inflammatory to pro-inflammatory pathways (3). Microorganisms and their products, including those in subgingival sites, can reach the amniotic fluid via hematogenous dissemination. They are then recognized by microbial pattern recognition receptors such as TLRs, signaling the pro-inflammatory pathways in placenta and also in fetal tissues. Furthermore, the signaling promoted by the periodontopathogens in gingival tissues and other sites of infection in the body contributes to the release of additional inflammatory mediators, which are spread systemically. This shift to an inflammatory environment not only activates labor but also leads to PE and restricts intrauterine growth.

## Effect of Periodontal Treatment on Gestational Alterations

The effect of periodontal treatment on preventing gestational alterations is another controversial issue. Given the evidences associating periodontitis and gestational complications, various intervention studies were performed in order to determinate the effect of periodontal treatment on pregnancy outcomes. Studies on the effect of non-surgical mechanical treatment in pregnant women with gingival inflammation but limited periodontal destruction indicated an improvement in clinical parameters and a decrease in GCF inflammatory markers, but resulted in no significant differences in gestational outcomes such as PT birth and/or low weight at birth (123, 124). One exception was a study conducted in Chile, where the treatment of pregnant women with gingivitis resulted in a reduction in PT and LBW (125).

Since the link between periodontitis and pregnancy outcomes seems to be the result of spread of bacteria and their factors to extra-oral sites, and the consequent inflammatory burden induced by the pathogens, it seems reasonable that the periodontal treatment would provide better benefits to pregnancy to patients with severe periodontitis than to those with only gingivitis. This issue seems especially relevant due to the age of women enrolled in large randomized populational studies (18–35 years), since severe periodontitis comprised a very low percentage of participants.

The prevalence of PT births was reduced in women with severe periodontitis that received periodontal treatment (125–128). These data contradict other reports, where no positive effects of periodontal treatment on pregnancy outcomes were observed (129–132).

Several systematic reviews with meta-analysis on the effect of maternal periodontal treatment and risk for PT birth and/or low birth weight did not indicate a decreased risk after treatment (69, 133–135). However, one review suggested a significant effect in reducing the risk of PT birth for scaling and root planning in pregnant women with periodontitis for groups with high risk of PT birth (136).

The conflicting data on the effect of periodontal therapy in preventing PT birth/low birth weight are probably due to differences in diagnostic criteria of periodontitis, and other conditions common to both such as smoking, age, and ethnicity. In some studies, only gingivitis patients were enrolled (125). Furthermore, differences in disease severity and extension may have accounted for differences in benefits in gestation outcomes promoted by treatment. For instance, in the study of Offenbacher et al. (126), which reported the beneficial effects of the periodontal treatment on gestational outcomes, periodontitis patients had at least two sites in different teeth with PD ≥5 mm and evidence of attachment loss. On the other hand, in another study where treatment provided no positive effect for gestation, periodontitis was classified as ≥3 mm of PD in ≥3 teeth (137).

The studies differed also in treatment strategies. After supra and subgingival mechanical treatment, patients received monthly periodontal maintenance (129), while in others follow-up was not mentioned (137). An early study conducted in Chile reported that the non-surgical periodontal treatment during pregnancy resulted in a reduction in PT and LBW from 10.11% in the control group to 1.84% in the test group (138). However, women with severe periodontitis in the treatment group were submitted to metronidazole and amoxicillin administration, which may have controlled not only the oral microbiota but may also have affected infecting agents in other sites.

Other differences refer to the control groups. In most studies, the control group received no periodontal treatment during pregnancy (123, 125, 128, 138), which was postponed after the delivery. One study had no control group for comparison (124), while in another (137), the control group received supragingival prophylaxis, which may have altered inflammation levels and proportions of pathogens, especially in shallow pockets.

Other limitations of these interventional studies include the pooling of periodontitis patients with different extension, severity, and progression rates in a single group. As discussed above, the microbiota and immune response differ between aggressive and chronic periodontitis patients, and possibly their effects on pregnancy outcomes may also differ.

The period of gestation for the periodontal intervention should also be taken into account when discussing the effect of periodontal treatment in gestational outcomes. One study reported that the periodontal treatment during the second trimester of pregnancy led to a reduction of 3.8 times in the risk of PT delivery compared with the group receiving treatment after birth (128). However, there are no data comparing the effect of treatment prior or at the first month of gestation with that performed at later stages, and most studies reported only that patients were selected before a certain gestational age, such as 14–20 weeks gestation. The placenta microbiome study showed that even placenta from periodontally healthy women may be colonized by oral organisms (28). Animal model studies indicated that gestational outcomes including low weight of the fetus are dependent on the period of infection, and an acute infection immediately before conception or at the middle of the gestation led to more severe damage to the fetus than infection at the beginning of gestation (119). Human and animal studies reported a microbial shift toward a more pathogenic microbiota in subgingival sites of periodontitis patients throughout the gestational period. Thus, one could assume that earlier periodontal treatment during pregnancy (or even before it) would be more beneficial than the treatment performed at the last trimester of gestation.

The effect of periodontal treatment on the prevention of PT delivery and other complications during pregnancy may be influenced by its effect on the bacterial load/composition and on the host inflammatory response. Infections in the lower genital tract have been associated with PT labor. Nevertheless, antibiotic treatment of asymptomatic women with bacterial vaginosis has not reduced the rate of PT delivery (3). Thus, other unknown genetic factors leading to increased susceptibility to the infection, to an abnormal response, and/or to PT labor may also play a role.

Gestation is a relatively short physiological process considering a chronic disease such as periodontitis. It is clear from most studies that periodontal treatment is able to improve clinical parameters of periodontitis (42, 123, 124, 131, 136); however, its effects on gestational outcomes involve several variables, making it hard to clearly evaluate the effect of periodontal treatment.

Despite the evidences associating PD with gestational outcomes, the effect of periodontal treatment in pregnancy is still not supported by scientific evidences. Periodontal treatment protocols providing the expected benefits for the mother and the fetus are still needed, and these should be tested in large randomized clinical trials. The periodontal treatment before pregnancy seems an option, since the severity of periodontitis and the virulence potential of the microbial community increase during pregnancy and the deleterious effects of the periodontal infection/inflammation to the fetus may occur even at very early stages of gestation.

## Conclusion

Periodontitis are associated with induction of premature births and other gestational complications, which may compromise the subject throughout life. Periodontal pathogens may be involved in this process, directly inducing fetal abnormalities, or inducing the inflammatory response and the contraction of myocytes, inducing prematurity. However, there are still no data on differences induced by different oral microbial communities. Furthermore, the current data had not provided conclusive answers for the effect of periodontal treatment in preventing PT deliveries and other gestational complications, and definitive intervention protocols were not established.

## Conflict of Interest Statement

The authors declare that the research was conducted in the absence of any commercial or financial relationships that could be construed as a potential conflict of interest.
